# Supplementary material for: OTU7B Modulates the Mosquito Immune Response to Beauveria bassiana Infection via Deubiquitination of the Toll Adaptor TRAF4
Source: Microbiol Spectr. 2022 Dec 20;11(1):e03123-22. doi: 10.1128/spectrum.03123-22 (PMC9927300; doi:10.1128/spectrum.03123-22)
Supplement: Supplemental file 1 — Supplemental material. Download spectrum.03123-22-s0001.pdf, PDF file, 1.7 MB [file spectrum.03123-22-s0001.pdf]

Supplemental Material

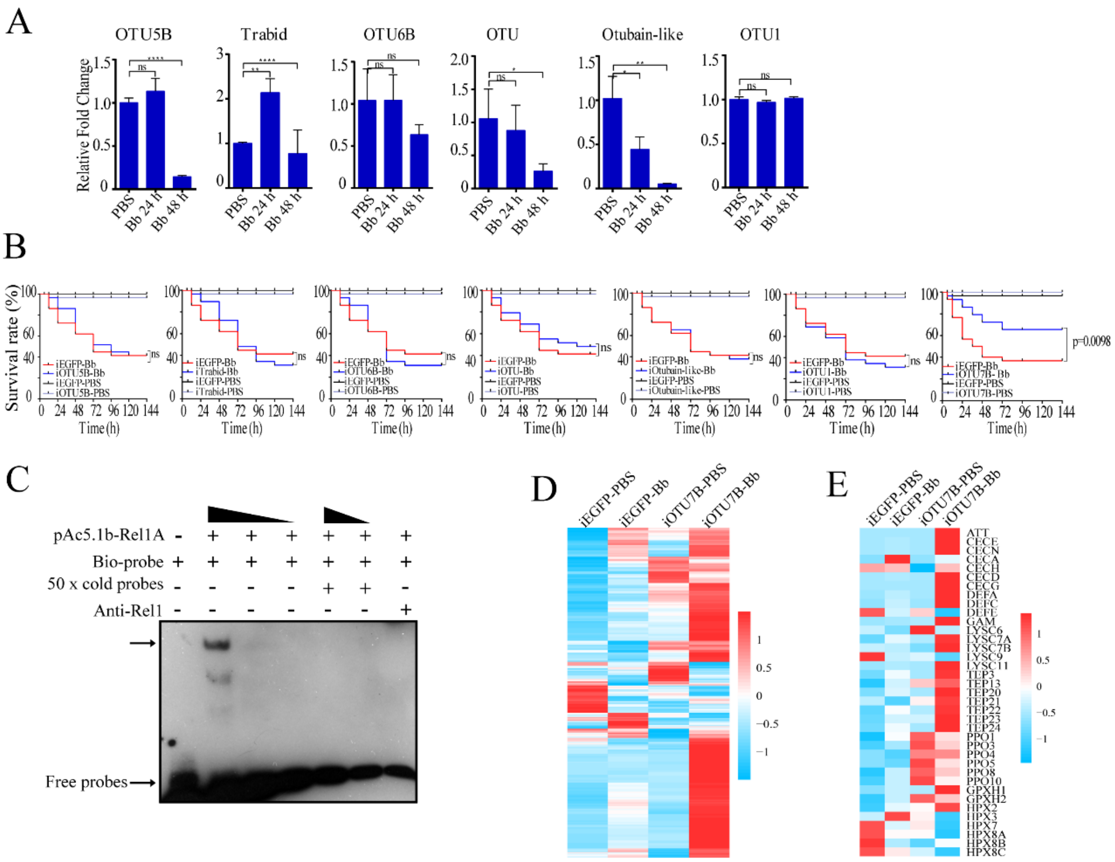

Figure S1 Response of OTU domain-containing proteins to fungal infection in mosquitoes. (A) qRT-PCR validation of transcription levels of genes encoding OTU domain-containing proteins at different time points after *B. bassiana* infection (Bb 24 h and Bb 48 h). The data are shown as the mean ± SEM. The data were normalized to the expression level of mosquitoes treated with PBS. **p* < 0.05, ***p* < 0.01, ****p* < 0.001, *****p* < 0.0001; statistically significant differences among samples were determined using the student's t test. (B) Survival assays were performed to determine the function of OTU-containing proteins in response to *B. bassiana* infection. Upon depletion of OTU7B, the mosquitoes were more resistant to fungal infection (p = 0.0098). EGFP dsRNA-treated mosquitoes (iEGFP) were used as control. Each experiment was performed in triplicate. (**C**) EMSA results showing the specific binding between Rel1A and *OTU7B* probe. The DNA probe containing putative NF-κB motif and its flanking regions from the promoter region of *OTU7B* was selected as probe. Different doses of nuclear protein extracts from Rel1A overexpressed Aag2 cells, 50 × unlabeled specific probes, and anti-Rel1 antibody were added as indicated. (D) Cluster analysis of differentially expressed immunity-related gene cohorts. EGFP dsRNA-treated mosquitoes were subsequently treated with PBS (iEGFP-PBS) or *B. bassiana* (iEGFP-Bb); OTU7B dsRNA-treated mosquitoes were later treated with PBS (iOTU7B-PBS) or *B. bassiana* (iOTU7B-Bb). (E) Heatmap of mosquito immune effector genes.

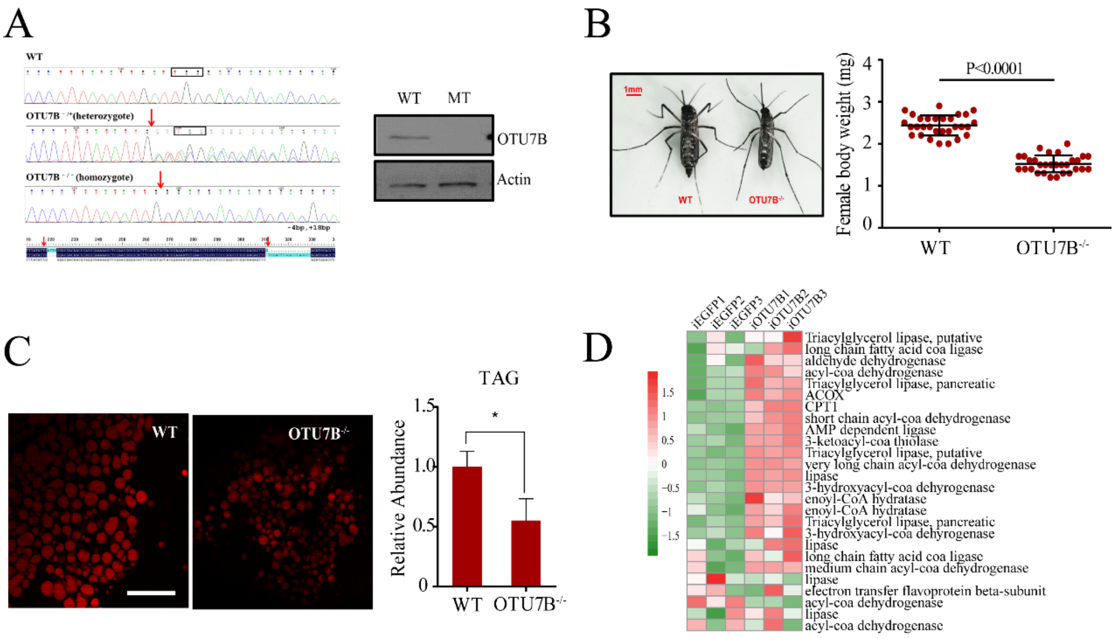

Figure S2 Transcriptome analysis of OTU7B modulation of mosquito immunity-related genes after fungal infection. (A) Representative chromatograms of sgRNA-targeted genomic region of the wild-type (WT), heterozygote (OTU7B$^{+/-}$), and homozygote (OTU7B$^{-/-}$) around the *OTU7B* gene are listed. The PAM sequence (NGG) is indicated by a square. Sequence alignment of WT and *OTU7B* knockout genotypes at the sgRNA-targeted genomic region. The genotype of the *OTU7B* KO line has a 4-bp deletion and an 11-bp insertion in the first exon. Immunoblotting results were used to examine the knockout effect of OTU7B using anti-OTU7B antibody. Anti-actin antibody was used as loading control. (B) Comparison of body size and body weight between OTU7B$^{-/-}$ and WT mosquitoes. Scale bar, 1 mm. (C) Comparison of lipid droplet size, TAG levels, and egg deposition between OTU7B$^{-/-}$ and WT. Lipid droplets in the FBs of OTU7B$^{-/-}$ and WT were detected using Nile red staining. At least three biological replicates for each (five fat bodies in each replicate) were used for TAG measurement. (D) Hierarchical cluster analysis of lipid-metabolism-related genes after knockdown of OTU7B.

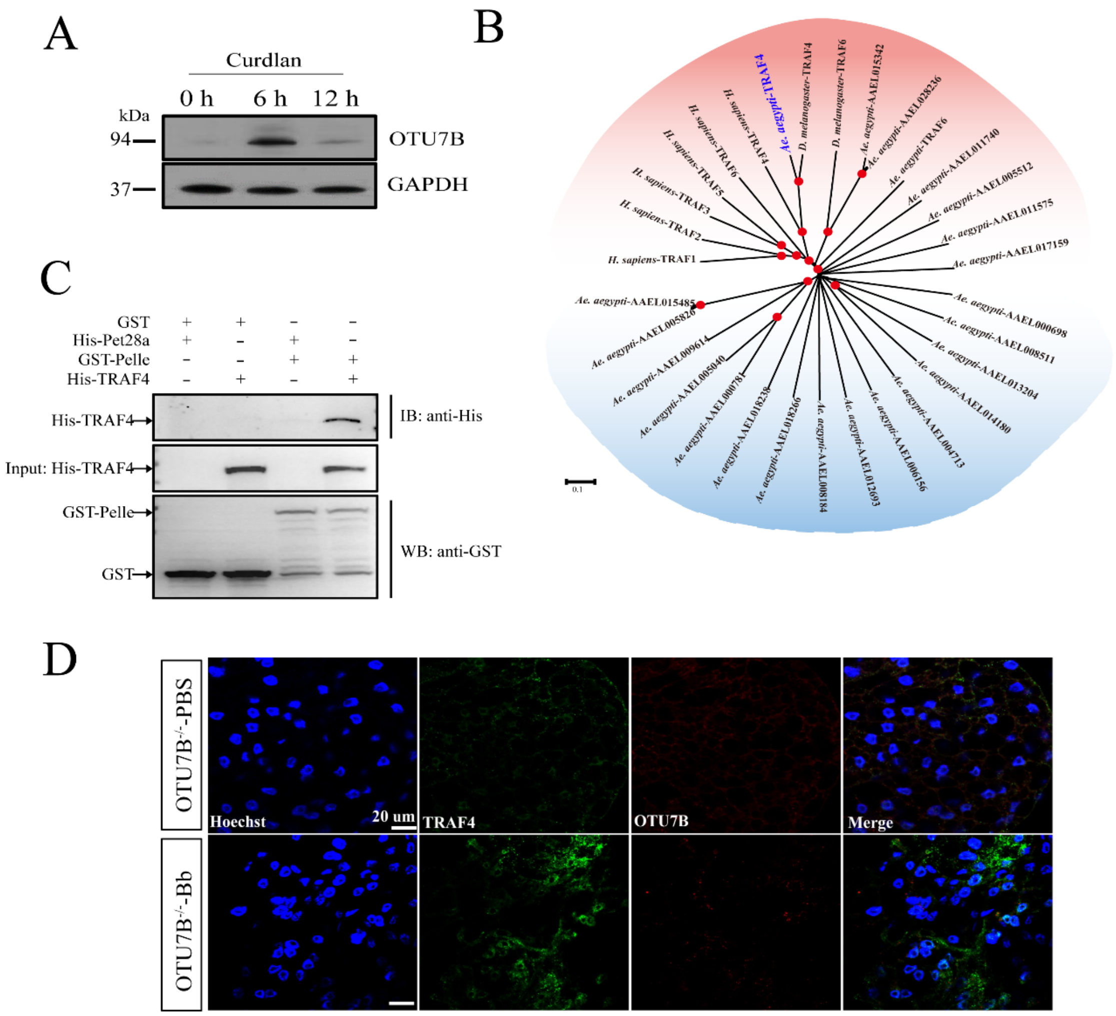

Figure S3 Overexpression of OTU7B in Aag2 cells and phylogenetic analysis of TRAFs. (A) Immunoblotting results showed the expression levels of OTU7B after Curdlan treatment for different durations. Anti-V5 antibody was used to detect OTU7B. Anti-GAPHD antibody was used as loading control. (B) TRAFs from *Ae. aegypti*, *D. melanogaster*, and *Homo sapiens* are shown. The TRAF4 of *Ae. aegypti* is marked blue. Red dots at nodes demonstrate bootstrap values above 90 out of 100. (C) GST pull-down assay between TRAF4 and Pelle. The interaction of His-TRAF4 with GST-Pelle was observed in the pull-down assay and confirmed by Immunoblotting with anti-GST and anti-His antibodies. (D) Immunofluorescence showing the expression of TRAF4 (red) in OTU7B$^{-/-}$mosquitoes after fungal infection. Scare bar, 20 µm.

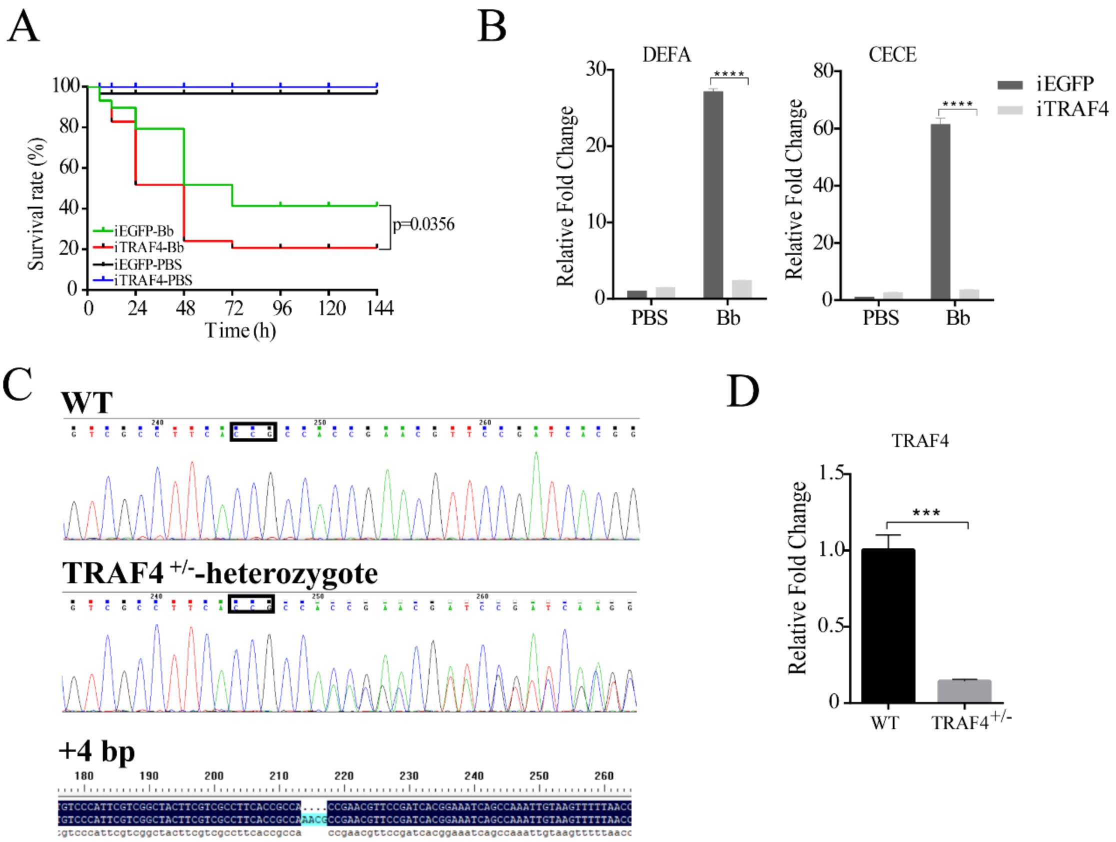

Figure S4 Response of TRAF4 to *B. bassiana* infection. (A) Survival rate of mosquitoes infected with *B. bassiana* significantly decreased with TRAF4 dsRNA injection (iTRAF4-Bb) compared with EGFP dsRNA injection (iEGFP-Bb). (B) qRT-PCR results showed the transcription levels of *DEFA* and *CECE* in iTRAF4 mosquito infection with fungal. (C) Representative chromatograms of sgRNA-targeted genomic region of the WT and heterozygote around gene *TRAF4* are listed. The PAM sequence (NGG) is indicated by a square. Sequence alignment of WT and selected *TRAF4* mutant genotypes at sgRNA-targeted genomic region. The genotype of the *TRAF4* mutant line has a 4-bp insertion in the first exon. (D) The mRNA level of TRAF4 in TRAF4$^{+/-}$ was determined using qRT-PCR.

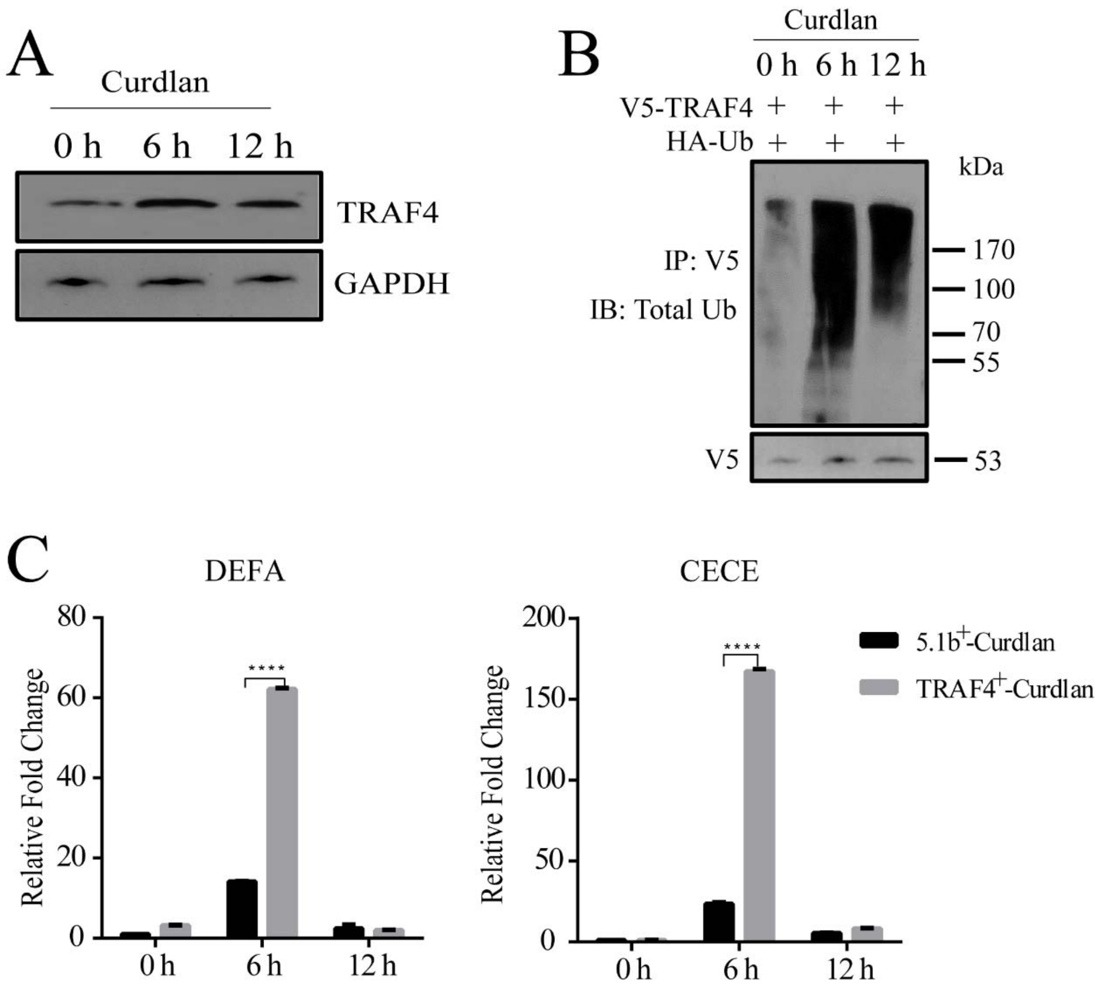

Figure S5 TRAF4 is ubiquitinated after fungal infection. (A) Immunoblotting results showed the expression level of TRAF4 in after Curdlan treatment for different durations. Anti-V5 antibody was used to detect TRAF4. Anti-GAPHD antibody was used as loading control. (B) Detection of ubiquitination of TRAF4 in Aag2 cells, which had been transfected with V5-TRAF4 and HA-Ub plasmids and then treated with Curdlan for different durations. Cell lysates were immunoprecipitated with anti-V5 antibody and analyzed by immunoblotting with anti-Total Ub antibody. Anti-V5 antibody was used as loading control. (C) The mRNA levels of *DEFA* and *CECE* were determined using qRT-PCR in Aag2 cells after infection with Curdlan for different time posts at transfection with TRAF4 ($TRAF4^{+}$-Curdlan) or pAC5.1b ($5.1b^{+}$-Curdlan) plasmids. The data are presented as the mean ± SEM. ****$p < 0.0001$. The experiments were repeated thrice, and the data were normalized to the expression level of cells treated with Curdlan for 0 h after transfection with pAC5.1b. Statistical analysis of the data was assessed using student's t test.

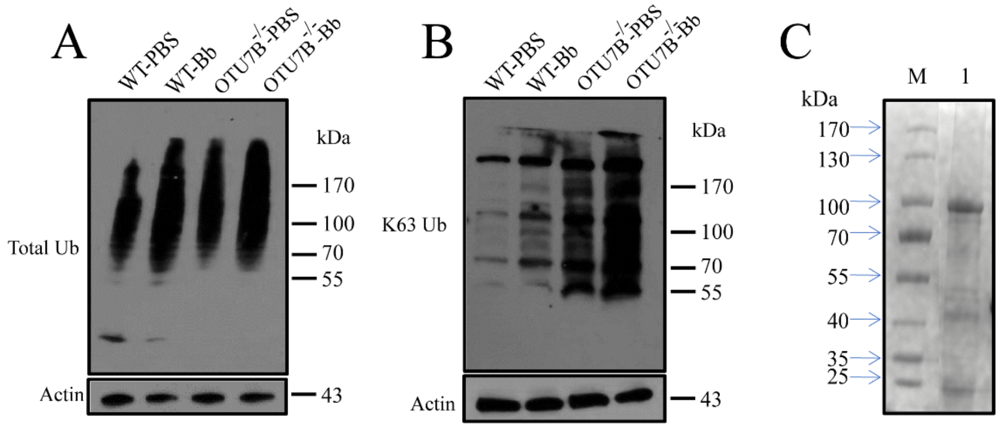

Figure S6 Detection of ubiquitination levels in mosquitoes after fungal challenge. (A-B) Ubiquitination levels of WT and OTU7B$^{-/-}$ mosquitoes were analyzed using immunoblotting with anti-total Ub antibody (A) and anti-K63 Ub antibody (B). Anti-actin antibody was used as loading control. (C) SDS-PAGE showed the purification of recombinant OTU7B. M, pre-stained protein ladder; 1, rOTU7B.

Table S1 List of differentially expressed immunity-related genes.

GeneID	iGFP-PBS	iGFP-Bb	iOTU7B-PBS	iOTU7B-Bb
AAEL000028	170.9986	232.7251	224.9956	321.4334
AAEL000037	22.64735	61.88216	36.64341	196.1229
AAEL000038	20.3069	20.79119	23.08645	31.53487
AAEL000059	25.44417	45.774	41.29639	89.7354
AAEL000074	66.89378	96.06625	84.66297	201.5227
AAEL000079	30.59356	29.62106	30.7517	28.28063
AAEL000086	0.445687	0.593634	0.762284	1.286815
AAEL000087	4.30673	10.18712	7.591253	18.88439
AAEL000099	26.55067	33.20101	36.49938	48.40409
AAEL000227	29.18193	32.26243	35.22256	34.54623
AAEL000234	18.2286	21.47334	23.00093	27.23087
AAEL000238	0.118015	0.158183	0.355619	0.119307
AAEL000256	6.376308	8.049256	7.59036	7.823993
AAEL000499	37.24436	34.84161	30.27338	34.46369
AAEL000508	0.796439	1.177714	1.17932	2.475447
AAEL000543	7.785063	11.3819	12.50846	15.561
AAEL000556	129.9061	113.361	134.1452	126.7785
AAEL000563	7.186531	4.59642	3.368359	2.927324
AAEL000589	46.55774	44.61103	46.92051	41.88538
AAEL000611	0.606306	56.02211	12.75526	1582.314
AAEL000621	1.04565	53.05059	12.64791	1401.017
AAEL000627	0	1.472646	0.067517	0.385718
AAEL000709	120.1428	139.1132	114.7953	179.5148
AAEL000749	19.18609	14.15498	24.54488	15.0631
AAEL000760	10.52967	13.07813	12.39748	13.36547
AAEL001077	3.922497	5.448404	6.951046	14.93192
AAEL001084	0.99546	1.486016	1.313637	4.211385
AAEL001163	6.965037	8.170409	8.189206	11.86893
AAEL001205	15.78895	15.70933	15.86597	17.46624
AAEL001713	0.440554	0.070094	0.052618	0.107669

AAEL001771	3.713653	4.607803	4.733685	4.306819
AAEL001794	23.16251	51.19743	35.95642	138.3736
AAEL001802	0.549971	0.391845	0.682768	1.028784
AAEL001914	2.833112	3.146966	3.554607	2.869663
AAEL002124	14.46217	15.33028	19.19512	20.30954
AAEL002126	2.923157	3.962206	3.699248	4.732046
AAEL002288	9.849179	20.41465	14.61887	25.54328
AAEL002301	53.60541	82.15203	68.33978	147.8233
AAEL002309	42.7193	45.73114	55.48356	62.20814
AAEL002524	4.899107	9.749212	11.2079	26.36145
AAEL002585	9.648606	26.21897	14.61639	69.61408
AAEL002590	13.85477	15.22825	14.41495	27.05663
AAEL002595	81.82233	148.6324	110.3542	309.5371
AAEL002601	36.07688	66.92883	50.59351	133.0191
AAEL002629	59.75574	78.61966	58.03135	92.12054
AAEL002704	33.50441	37.14338	40.5416	47.03936
AAEL002720	86.97297	119.4708	123.0353	221.6065
AAEL002730	6.356528	7.296572	5.919452	9.341274
AAEL002767	4.074483	4.715603	4.819934	10.74024
AAEL003104	23.05856	23.10445	22.87864	26.39726
AAEL003119	20.90882	26.22098	25.15188	28.26269
AAEL003156	0.07017	0.093146	0.053098	0.677905
AAEL003182	52.62013	52.51737	56.15708	50.61071
AAEL003243	45.24043	71.2265	49.69918	87.54444
AAEL003253	3.26152	8.518512	3.87011	8.651834
AAEL003294	1.634635	4.577526	11.95925	17.62684
AAEL003325	58.1065	60.40669	61.65168	60.98727
AAEL003371	64.96453	58.69496	56.57032	57.30219
AAEL003389	0.367559	0.507265	1.058466	123.4239
AAEL003439	2.314822	1.786462	1.352863	5.887247
AAEL003444	2.651417	3.335988	1.94779	3.920413
AAEL003503	92.85747	100.5499	93.55174	117.7883

AAEL003505	22.77474	26.99141	20.59236	29.0038
AAEL003541	36.32027	33.93234	36.8307	38.74856
AAEL003614	5.886272	16.71928	8.771516	33.00724
AAEL003625	186.0033	190.661	206.4622	229.1463
AAEL003628	4.693388	5.460891	4.302955	6.44874
AAEL003631	4.475756	7.72585	6.986546	8.76675
AAEL003632	0.533053	1.925573	0.94921	9.341956
AAEL003642	18.32785	29.69169	25.88468	48.30296
AAEL003686	74.60342	121.3074	94.79241	193.9666
AAEL003697	18.13143	27.84651	20.35036	36.81737
AAEL003723	22.14247	45.7276	38.74411	386.3816
AAEL003832	1.191094	113.1507	18.47094	784.7947
AAEL003841	2.649617	268.9933	51.67084	3105.844
AAEL003844	165.8026	168.7957	178.0795	185.7453
AAEL003849	378.9596	330.1237	349.1937	296.9075
AAEL003889	32.37612	41.29341	39.30693	69.74427
AAEL003933	1.34981	1.479747	1.520627	1.923012
AAEL004000	0.976611	1.43073	1.144381	1.237285
AAEL004112	515.2769	450.8348	467.6062	489.1281
AAEL004120	51.41482	44.86236	58.15236	87.53776
AAEL004150	0.015712	0.28134	0	0.080599
AAEL004156	19.93319	19.51477	21.75635	42.77208
AAEL004386	171.4379	159.1001	154.7767	144.5284
AAEL004388	124.6858	114.6841	114.8483	104.2966
AAEL004390	280.4849	258.2352	252.7399	262.9202
AAEL004401	97.07267	87.52519	90.09731	81.44565
AAEL004522	398.1207	434.4696	457.0295	1211.827
AAEL004540	24.12045	26.0599	25.02936	31.56397
AAEL004823	181.5617	175.762	199.1037	182.319
AAEL004979	3.302087	3.157623	2.643999	3.771063
AAEL005064	43.3869	60.00103	49.47094	70.77955
AAEL005093	6.554955	13.32122	13.18692	74.61112

AAEL005194	0.570908	1.039167	1.864777	3.978115
AAEL005293	60.14758	30.25353	37.59688	49.97006
AAEL005339	23.60267	24.27055	22.47775	24.13628
AAEL005359	12.62882	15.05676	13.43429	13.02516
AAEL005374	1.224758	1.724083	0.418376	0.641074
AAEL005416	30.36996	34.4716	31.29147	28.38972
AAEL005431	3.972826	9.19583	4.251395	26.31542
AAEL005482	69.74113	91.8524	84.32993	90.79113
AAEL005641	11.1568	26.98667	13.84804	211.7061
AAEL005665	43.97228	48.92125	48.25156	60.01118
AAEL005670	9.61821	11.23383	9.761673	15.01537
AAEL005673	79.85238	124.5515	122.2177	237.0654
AAEL005718	0.383135	1.150631	0.18399	0.312654
AAEL005748	2.718779	15.30936	8.494622	27.28389
AAEL005792	1.117025	4.612472	1.872225	43.20443
AAEL005800	0.965817	2.049196	1.429563	7.099425
AAEL005956	10.26636	8.212644	9.122447	8.98868
AAEL005963	4.381605	3.381403	2.824745	3.504582
AAEL005979	3.246425	3.754402	3.405169	5.700697
AAEL005988	4.081207	3.8458	5.08568	4.066748
AAEL006032	40.28683	42.07631	35.81369	32.85464
AAEL006161	9.689218	13.14297	11.65716	15.04423
AAEL006168	7.797174	12.93564	9.862101	18.78123
AAEL006212	0.128096	0.125299	0.191063	0.482467
AAEL006271	277.2536	247.9794	240.8624	225.6376
AAEL006355	1.389601	1.116121	2.184792	2.090449
AAEL006361	0.110934	0.320985	0.667271	0.688817
AAEL006610	26.99709	25.12005	27.39273	22.65123
AAEL006633	35.02055	30.61642	31.28798	29.72102
AAEL006674	122.8577	175.1445	166.3625	232.4581
AAEL006691	14.16842	10.66652	14.3275	17.84137
AAEL006699	16.22839	15.04833	15.36616	26.48732

AAEL006700	3.1281	4.815035	4.797573	5.232524
AAEL006702	16.75963	16.3173	18.20116	24.59431
AAEL006704	158.2224	90.47017	129.9741	302.6544
AAEL006794	21.97564	22.58142	20.98078	30.77556
AAEL006949	14.70999	14.92018	13.91735	15.4245
AAEL006973	6.989936	7.530515	6.399542	7.193653
AAEL007065	488.2557	479.2704	474.5594	495.5025
AAEL007420	15.29364	15.18788	18.25999	14.9794
AAEL007593	30.91919	28.66492	37.31969	40.1453
AAEL007597	39.71293	41.14147	34.61555	48.77948
AAEL007613	5.40563	5.904335	5.155349	7.002226
AAEL007619	5.030485	5.725687	8.038051	7.578317
AAEL007624	6.768327	8.2566	9.164331	14.01527
AAEL007626	9.590079	12.95123	10.71753	32.74141
AAEL007642	8.2614	9.503736	8.144202	6.91715
AAEL007696	71.59275	74.15189	62.91595	75.51446
AAEL007765	69.96813	74.74224	69.95913	104.2919
AAEL007768	4.637137	5.244119	6.322707	6.469012
AAEL007796	10.60062	12.0018	14.86082	16.56297
AAEL007942	20.08917	16.97366	22.81047	35.9401
AAEL007992	2.879931	7.58891	4.053603	22.97387
AAEL007993	1.447639	1.159764	1.509557	10.22791
AAEL008364	39.74888	44.55295	38.01658	59.40289
AAEL008397	24.7323	28.85057	36.10668	32.58106
AAEL008596	3.950639	6.663009	4.167722	13.84468
AAEL008607	25.60153	50.65548	36.57534	105.4813
AAEL008634	71.23517	62.57911	61.56634	62.73085
AAEL008646	1.773777	6.568303	7.38209	69.45098
AAEL008668	3.362598	4.106101	3.804211	6.823851
AAEL009051	36.79289	32.35075	31.45262	32.72272
AAEL009074	189.349	212.5433	190.7428	248.8685
AAEL009192	30.15018	37.51844	37.94695	44.82542

AAEL009384	0.222158	0.880703	3.259233	4.759753
AAEL009423	28.13271	32.75546	32.08163	41.90833
AAEL009432	7.067412	4.887223	6.774168	5.448832
AAEL009436	5.067719	3.931656	3.630748	3.972601
AAEL009474	17.82553	24.25029	24.89056	139.2823
AAEL009531	5.568252	5.058122	5.927147	5.928079
AAEL009670	110.0735	95.73213	97.01831	91.10742
AAEL009692	44.95705	48.28593	50.97456	52.90311
AAEL009760	129.0911	150.1349	182.5927	155.3491
AAEL009842	43.7168	41.62185	34.09248	42.21568
AAEL009850	5.278681	4.83159	4.7833	10.33628
AAEL009906	37.35314	38.14583	40.13942	35.13945
AAEL010100	27.16913	43.27885	36.43822	78.6297
AAEL010147	8.504234	8.344079	9.099625	8.624974
AAEL010171	29.38225	24.54142	27.31363	65.0398
AAEL010270	9.354897	9.601848	6.779632	18.31393
AAEL010655	0.762573	0.810979	1.100793	0.794414
AAEL010769	8.329155	11.47283	13.98789	21.70802
AAEL010773	9.8832	22.04248	16.96883	39.08222
AAEL010831	1.326052	1.920447	2.673065	2.033553
AAEL011070	35.66721	45.48828	46.30187	65.7781
AAEL017265	10.142225	13.023263	15.04227	36.290004
AAEL011222	11.49331	11.41923	13.21831	12.5095
AAEL011400	15.679	18.5424	20.36359	18.29946
AAEL011404	1.077056	1.997896	2.725245	2.663782
AAEL011408	11.37568	23.76215	15.55235	30.00679
AAEL011446	13.87063	22.8609	18.07617	37.27156
AAEL011453	9.184686	38.34209	13.17015	71.02069
AAEL011455	21.98726	65.88271	29.26955	90.51766
AAEL011562	15.15323	16.17973	14.02024	16.05305
AAEL011608	82.45836	68.53352	95.91165	93.43306
AAEL011610	2.1546	11.08085	8.187499	65.30947

AAEL011616	0.743443	1.380256	0.779495	1.351745
AAEL011621	1.171954	1.263572	1.352588	4.54779
AAEL011622	13.71842	19.09964	16.37079	85.02901
AAEL011634	0.320964	0.151721	0.388441	0.431951
AAEL011763	1.619129	1.991894	3.144558	2.543737
AAEL011764	0.856248	1.217361	1.983903	1.395983
AAEL011777	19.27351	24.13151	20.12728	38.23928
AAEL011991	9.687596	12.96872	13.51639	24.85716
AAEL012069	90.29364	99.56419	96.37091	149.026
AAEL012135	15.45545	12.61046	12.52577	17.75839
AAEL012267	5.159702	5.842681	6.261007	6.772644
AAEL012353	1.937962	7.684275	6.315392	35.51647
AAEL012380	12.19201	14.44005	11.97383	15.52511
AAEL012510	38.38311	39.04791	38.82762	42.74144
AAEL012712	19.76464	33.69698	21.01303	43.51429
AAEL012713	56.01842	66.18319	66.00545	70.13004
AAEL012723	13.62542	10.9437	13.16074	13.33992
AAEL012785	3.541917	5.300254	6.019535	12.44076
AAEL012941	6.18179	8.378824	11.41772	10.91717
AAEL013171	23.4379	35.55576	37.78966	53.79348
AAEL013245	2.03043	6.533268	3.45867	19.78089
AAEL013407	144.6232	148.0229	157.0971	145.943
AAEL013413	3.326612	3.661331	4.201928	4.877781
AAEL013417	0.591188	1.380149	1.077775	0.953738
AAEL013433	15.04985	19.01922	19.36157	23.9139
AAEL013434	4.467753	6.873125	5.44698	6.865342
AAEL013492	0.989264	0.883471	2.615164	1.751776
AAEL013496	0.484692	0.8129	1.346009	1.071888
AAEL013498	0.429925	0.790575	1.090667	0.857406
AAEL013501	0.425278	0.35977	0.752233	0.739183
AAEL013936	112.8457	152.9614	135.9456	232.3558
AAEL014078	3.867355	4.68592	6.400163	10.11551

AAEL014079	4.28201	5.479198	5.227495	5.646418
AAEL014137	2.293706	2.452135	3.073258	3.762191
AAEL014138	2.5784	12.41713	4.129772	23.586
AAEL014139	1.058656	3.259047	1.502853	3.503836
AAEL014141	10.82793	12.23102	8.162245	14.36328
AAEL014348	4.979299	4.540481	5.388478	8.305344
AAEL014349	9.854318	19.34941	14.39068	71.25186
AAEL014354	3.731954	6.074165	5.360318	6.794128
AAEL014356	5.070936	3.945397	3.263198	3.744157
AAEL014367	4.165154	5.009227	5.282293	5.268357
AAEL014382	2.383667	4.131013	7.730711	20.3116
AAEL014390	0.145101	0.784585	0.124513	4.433027
AAEL014548	62.79262	56.77991	70.00791	60.82158
AAEL014640	19.56328	17.06776	26.36674	27.09535
AAEL014642	118.2467	122.391	102.4172	103.5636
AAEL014658	5.497104	3.787705	3.476099	7.235987
AAEL014755	5.926615	5.311611	11.66753	21.88544
AAEL014837	0.563952	0.78448	1.120315	0.97115
AAEL014896	17.07812	19.12034	17.34693	15.76335
AAEL014950	8.750361	9.464286	12.38504	22.31334
AAEL014980	19.61979	34.8544	28.85406	58.87999
AAEL015136	50.91341	26.30189	22.24477	36.88696
AAEL015404	82.87853	80.05642	71.72237	127.2505
AAEL015439	0.805115	0.216852	0.475153	0.453824
AAEL015515	0.082902	26.96151	16.17517	1843.867
AAEL017023	13.9986	39.90207	24.13576	99.84813
AAEL017211	701.0171	691.8901	584.2305	690.3553
AAEL017325	49.40004	66.42013	73.83167	161.4051
AAEL017515	29.04802	27.60057	28.04737	30.92027
AAEL018349	0.476592	7.677011	1.466501	189.3596

Table S2 Repertoire of common upregulated immunity-related genes in iGFP-Bb, iOTU7B, and iOTU7B-Bb.

GeneID	iGFP-PBS	iGFP-Bb	iOTU7B-PBS	iOTU7B-Bb	Gene name
AAEL011610	2.1546	11.08085	8.187499	65.30947	CTLGA7
AAEL012353	1.937962	7.684275	6.315392	35.51647	CTL15
AAEL011404	1.077056	1.997896	2.725245	2.663782	CTL19
AAEL002524	4.899107	9.749212	11.2079	26.36145	CTL24
AAEL000543	7.785063	11.3819	12.50846	15.561	CTLMA11
AAEL014382	2.383667	4.131013	7.730711	20.3116	CTLMA14
AAEL009384	0.222158	0.880703	3.259233	4.759753	FREP5
AAEL003294	1.634635	4.577526	11.95925	17.62684	FREP3
AAEL008646	1.773777	6.568303	7.38209	69.45098	FREP10
AAEL005194	0.570908	1.039167	1.864777	3.978115	FREP26
AAEL013417	0.591188	1.380149	1.077775	0.953738	FREP24
AAEL000508	0.796439	1.177714	1.17932	2.475447	FREP15
AAEL006361	0.110934	0.320985	0.667271	0.688817	SCRC2
AAEL002288	9.849179	20.41465	14.61887	25.54328	CLIPA4
AAEL002585	9.648606	26.21897	14.61639	69.61408	CLIPA11
AAEL014349	9.854318	19.34941	14.39068	71.25186	CLIPB15
AAEL000059	25.44417	45.774	41.29639	89.7354	CLIPB19
AAEL013245	2.03043	6.533268	3.45867	19.78089	CLIPB28
AAEL000037	22.64735	61.88216	36.64341	196.1229	CLIPB35
AAEL003632	0.533053	1.925573	0.94921	9.341956	CLIPB39
AAEL003614	5.886272	16.71928	8.771516	33.00724	CLIPB40
AAEL003631	4.475756	7.72585	6.986546	8.76675	CLIPB41
AAEL005093	6.554955	13.32122	13.18692	74.61112	CLIPB46
AAEL005792	1.117025	4.612472	1.872225	43.20443	CLIPE8
AAEL010773	9.8832	22.04248	16.96883	39.08222	CLIPE10
AAEL005800	0.965817	2.049196	1.429563	7.099425	CLIPE11
AAEL012785	3.541917	5.300254	6.019535	12.44076	CLIPE13
AAEL005748	2.718779	15.30936	8.494622	27.28389	CBSP

AAEL006700	3.1281	4.815035	4.797573	5.232524	CUBSP3
AAEL014138	2.5784	12.41713	4.129772	23.586	SRPN16
AAEL014980	19.61979	34.8544	28.85406	58.87999	SRPN24
AAEL005673	79.85238	124.5515	122.2177	237.0654	SRPN28
AAEL000627	0	1.472646	0.067517	0.385718	CECA
AAEL015515	0.082902	26.96151	16.17517	1843.867	CECG
AAEL000611	0.606306	56.02211	12.75526	1582.314	CECE
AAEL003841	2.649617	268.9933	51.67084	3105.844	DEFA
AAEL003832	1.191094	113.1507	18.47094	784.7947	DEFC
AAEL018349	0.476592	7.677011	1.466501	189.3596	CECD
AAEL000621	1.04565	53.05059	12.64791	1401.017	CECN
AAEL003723	22.14247	45.7276	38.74411	386.3816	LYSC11
AAEL001794	23.16251	51.19743	35.95642	138.3736	TEP20
AAEL000087	4.30673	10.18712	7.591253	18.88439	TEP22
AAEL017023	13.9986	39.90207	24.13576	99.84813	TEP24
AAEL013171	23.4379	35.55576	37.78966	53.79348	HPX2
AAEL013498	0.429925	0.790575	1.090667	0.857406	PPO1
AAEL013496	0.484692	0.8129	1.346009	1.071888	PPO8

Table S3 Repertoire of proteins identified from cells treated with DMSO after pAC-5.1b-OTU7B transfection.

Accession	Description	Mass	Score	Matches	Sequences	emPAI	Coverage
AAEL012175	ATP synthase alpha subunit mitochondrial	59526	283	17(10)	13(9)	0.62	22%
AAEL002827	ATP synthase beta subunit	53878	177	8(5)	7(5)	0.43	17%
AAEL017096	Elongation factor 1-alpha	50784	138	5(5)	5(5)	0.37	15%
AAEL008658	LRIM16: leucine-rich immune protein	79910	110	2(2)	2(2)	0.08	3%
AAEL000501	histone H4	11392	104	4(4)	4(4)	1.87	40%
AAEL023386	hypothetical protein	82587	83	5(4)	1(1)	0.08	0%
AAEL017349	hypothetical protein	72356	35	2(1)	2(1)	0.05	3%
AAEL017495	GPROR95: Odorant receptor	49114	33	3(1)	1(1)	0.07	1%
AAEL009883	26S protease (S4) regulatory subunit	67460	32	1(1)	1(1)	0.05	1%
AAEL017994	hypothetical protein	138567	30	1(1)	1(1)	0.02	0%
AAEL006511	ubiquitin	15005	28	2(1)	2(1)	0.23	11%
AAEL001673	actin	42149	26	3(1)	2(1)	0.08	3%
AAEL013366	hypothetical protein	108095	15	1(1)	1(1)	0.03	0%

Table S4 Repertoire of proteins identified from cells treated with DMSO after pAC-5.1b-OTU7B transfection.

Accession	Description	Mass	Score	Matches	Sequences	emPAI	Coverage
AAEL012175	ATP synthase alpha subunit mitochondrial	59526	283	17(10)	13(9)	0.62	22%
AAEL002827	ATP synthase beta subunit	53878	177	8(5)	7(5)	0.43	17%
AAEL017096	Elongation factor 1-alpha	50784	138	5(5)	5(5)	0.37	15%
AAEL008658	LRIM16: leucine-rich immune protein	79910	110	2(2)	2(2)	0.08	3%
AAEL000501	histone H4	11392	104	4(4)	4(4)	1.87	40%
AAEL023386	hypothetical protein	82587	83	5(4)	1(1)	0.08	0%
AAEL017349	hypothetical protein	72356	35	2(1)	2(1)	0.05	3%
AAEL017495	GPROR95: Odorant receptor	49114	33	3(1)	1(1)	0.07	1%
AAEL009883	26S protease (S4) regulatory subunit	67460	32	1(1)	1(1)	0.05	1%
AAEL017994	hypothetical protein	138567	30	1(1)	1(1)	0.02	0%
AAEL006511	ubiquitin	15005	28	2(1)	2(1)	0.23	11%
AAEL001673	actin	42149	26	3(1)	2(1)	0.08	3%
AAEL013366	hypothetical protein	108095	15	1(1)	1(1)	0.03	0%

Table S5 Primers used in this article.

**Primers for knock-out**	
OTU7B-KO1	GAAATTAATACGACTCACTATAGGCCCGGCAAGAGTT CGAGAGTTTTAGAGCTAGAAATAGC
OTU7B-KO2	GAAATTAATACGACTCACTATAGACATCCCCTTTATAT TGATGTTTTAGAGCTAGAAATAGC
OTU7B-KO3	GAAATTAATACGACTCACTATAGTCTACACTTAACTCG CTGGGTTTTAGAGCTAGAAATAGC
TRAF4-KO1	GAAATTAATACGACTCACTATAGCTGCCAATTACACC GCCTCGTTTTAGAGCTAGAAATAGC
TRAF4-KO2	GAAATTAATACGACTCACTATAGTGGTCGACTTGTTCC TCGGGTTTTAGAGCTAGAAATAGC
TRAF4-KO3	GAAATTAATACGACTCACTATAGTGATCGGAACGTTC GGTGGGTTTTAGAGCTAGAAATAGC
KO	AAAAGCACCGACTCGGTGCCACTTTTTCAAGTTGATA ACGGACTAGCCTTATTTTAACTTGCTATTTCTAGCTCT AAAAC
OTU7B-KO identification	AGCTGCATGGATGCTCAAGTT
	CGCCTCCAGCGAGTTAAGTGT
TRAF4-KO identification	CCGATGGTAGTTTAGGAGGA
	GAGTGGTGGGGAATTGTTTG
TRAF4-KO identification2	CCGATGGTAGTTTAGGAGGA
	GTGATCGGAACGTTCGGCGTT
**Primers for RNAi**	
OTU5B	TATTAGATCCGTACAAAGCC
	TATTGAACCGAACAAGAAGT
	TAATACGACTCACTATAGGGTATTAGATCCGTACAAA GCC
	TAATACGACTCACTATAGGGTATTGAACCGAACAAGA AGT
Trabid	AGAAGCCTACAGCCTCAAAG
	TCTCCGCTATCCCAATACAA
	TAATACGACTCACTATAGGGAGAAGCCTACAGCCTCA AAG

	TAATACGACTCACTATAGGGTCTCCGCTATCCCAATACAA
OTU6B	GAAGCGAAACAGCCAAAGGT
	ATGTGCCGATGGTAGGTCAG
	TAATACGACTCACTATAGGGGAAGCGAAACAGCCAAAGGT
	TAATACGACTCACTATAGGGATGTGCCGATGGTAGGTCAG
OTU	ACGGTGGATGATTTGATTAC
	TTATAGCAGGATGCGTAGGA
	TAATACGACTCACTATAGGGACGGTGGATGATTTGATTAC
	TAATACGACTCACTATAGGGTTATAGCAGGATGCGTAGGA
Otubain-like	ATGAGCACTTCGGACAGCAC
	TCAAGCGTAGATAAACCACC
	TAATACGACTCACTATAGGGATGAGCACTTCGGACAGCAC
	TAATACGACTCACTATAGGGTCAAGCGTAGATAAACCACC
OTU1	AGAAGCAACTGAGCGAGGAG
	GGAACATAGTTTGCGGAGGT
	TAATACGACTCACTATAGGGAGAAGCAACTGAGCGAGGAG
	TAATACGACTCACTATAGGGGGAACATAGTTTGCGGAGGT
OTU7B	TAAGAGTGGGCAGCATAAGA
	GTTTGGCACAGTTGGAATAG
	TAATACGACTCACTATAGGG TAAGAGTGGGCAGCATAAGA
	TAATACGACTCACTATAGGG GTTTGGCACAGTTGGAATAG
Rel1A	CGCACCCGTTTAACTTCATT
	TGGACCATTAGCGATTTCAG
	TAATACGACTCACTATAGGG CGCACCCGTTTAACTTCATT

	TAATACGACTCACTATAGGG TGGACCATTAGCGATTTCAG
TRAF4	ATCCACCGTGCCAAAGATTG
	GGTCCGTTGCCATTGAGAAA
	TAATACGACTCACTATAGGG ATCCACCGTGCCAAAGATTG
	TAATACGACTCACTATAGGG GGTCCGTTGCCATTGAGAAA
**Primers for clone**	
OTU7B-pET28a	GCATGACTGGTGGACAG ATGGGAAAACAACACATCCG
	CTAGTTATTGCTCAGCGG GATGTTGTTCTCCTGGCAGC
OTU7B-pAC5.1b-V5	GTGTGGTGGAATTCTGCA ATGGGAAAACAACACATCCG
	GCCACTGTGCTGGATATC GATGTTGTTCTCCTGGCAGC
TRAF4-pET28a	GCATGACTGGTGGACAG CAGTGGACCAAAACGCTCAG
	CTAGTTATTGCTCAGCGG CTTGCTGGGATCCACTTTGAC
TRAF4-pAC5.1b-V5	GTGTGGTGGAATTCTGCA CAGTGGACCAAAACGCTCAG
	GCCACTGTGCTGGATATC CTTGCTGGGATCCACTTTGAC
TRAF4-mutation	CGCTCCTGTGGAAAATCACCGACTGGTCAGCTCGAAT GGTCGAAG
	GATTTTCCACAGGAGCGTGCCGGTGTAGTTGGTACTG AGTTTGGC
pAC5.1b-HA	TACGACGTCCCAGACTACGCTCGTACCGGTCATCATCA CCATCACCATTGAG
	TAGTCTGGGACGTCGTATGGGTATTCGAAGGGCCCTCT AGACTCGAGCGGCCGCCAC
OTU7B-pAC5.1b-Flag	ACAAGGATGACGACGATAAGCGTACCGGTCATCATCA CCATCACCATG
	ATCGTCGTCATCCTTGTAATCTTCGAACCGCGGGCCCT CTAGACTCGAG
OTU7B-PGL4.10	CCGGTACCTGAGCTCGCTGGTGCAGCCGTCTTTGAGTA
	CTTGATATCCTCGAGGCTCAGTTTCCGCCACGAGGTTA

Rel1A-pAc5.1b-V5	GTGTGGTGGAATTCTGCAATGTCCTTCCCCACAAAACTT
	GCCACTGTGCTGGATATCGACATCAGCGCTGTAAGCC
Rel1B-pAc5.1b-V5	GTGTGGTGGAATTCTGCAATGCTCAGTCACAATCTATCAG
	GCCACTGTGCTGGATATCCTTGTTCATGTTGTTGACAGTG
Ubiquitin-pAC5.1b-HA	ATGCGGTACCATGCAGATCTTCGTCAAGAC
	ATGCGCGGCCGCACCACCACGAAGACGCAGCA
K6 mutant	CCATGCAGATCTTCGTCAGGACCCTTACGGGCAAGACCATCACCCTTG
	GACGAAGATCTGCATGGTACCCCGATCCGGGTCTCTGAATAAACGGGC
K11 mutant	CCATGCAGATCTTCGTCAAGACCCTTACGGGCAGGACCATCACCCTTG
K27 mutant	GGACACGATCGAAAATGTGAGGGCTAAGATCCAGGACAAGGAAGGAAT
	CATTTTCGATCGTGTCCGAAGGCTCGACCTCAAGGGTGATGGTCTTGC
K29 mutant	GGACACGATCGAAAATGTGAAGGCTAGGATCCAGGACAAGGAAGGAAT
K33 mutant	GGACACGATCGAAAATGTGAAGGCTAAGATCCAGGACAGGGAAGGAAT
K48 mutant	GAAGGAATTCCCCCAGATCAGCAGCGTTTGATCTTCGCCGGAAGGCAGC
	TCTGGGGGAATTCCTTCCTTGTCCTGGATCTTAGCCTTCACATTTTCG
K63 mutant	ACCCTGTCCGACTACAACATCCAGAGGGAATCCACTCTCCATCTGGTG
	TTGTAGTCGGACAGGGTGCGGCCATCTTCCAGCTGCTTTCCGGCGAAG
	GTGTGGTGGAATTCTGCAATGTCAGCTGAACCATCACA
Pelle-pAc5.1b-V5	GCCACTGTGCTGGATATCTGGTCGAGGTTTTTGGGGAA
**Primers for qRT-PCR**	
OTU5B	ATGAGCCGCTACGGTTGTCG

	TCGGACTGCATCTGCCATTT
Trabid	CTCGTCGCTGTCTACAATGC
	TCTTCTTCGCCAGTACTTCG
OTU6B	CGGAGGATTTGGGAGGTGTT
	CTTCTGCCTTGATCTGTGCC
OTU	GCTTATGCTGCTCCTGGTCC
	CAGGAACTGGTGGCGGAAGC
Otubain-like	GCAGGGATATTCAGATTACG
	ATGTGGTCCGACTCCTTGTA
OTU1	TTACCAACGGGGACACGCTA
	TGCTTGAGCAAGATCCCACC
OTU7B	GGATTGGCAGTAAGAGTGGG
	TCTTCCTGGTATTCGTGTCG
TRAF4	CCGAACCGATCTACTGTGAA
	TCCGTGAGCGGATAGCGTAT
Rel1A	CAATGTGCCGGGAGAAGCTA
	TGTTGCCGTAAAGTGGGGAT
Rel1B	CCGTGATGGTTCACATTCAG
	CTGTTGAACTGGGACCGTTT
DEFA	TCCCTCACTGTCATTTGTTT
	CAGGCGGAAGTTCTCCACGG
CECE	TTCCGACTCCTTCCAATTTC
	TTCCGACTCCTTCCAATTTC
ATT	TTGGCAGGCACGGAATGT
	CGCCCAGACCTAAGGAAGC
TEP20	GAACGACCATTAGCAACCTG
	TGCTTTGGTCCTCACATCTT
Lys11	AAAGCTCTACTCGCTCAAGG
	TTGCGATTGTTGTTCTTCTT
**Probes for EMSA**	
Probe-1	TGGATAATGGCGGTCCCCCAAGGAAC
	GTTCCTTGGGGGACCGCCATTATCCA

Probe-2	AAAAGTTTGGGAACCTATGGCTTAGG
	CCTAAGCCATAGGTTCCCAAACTTTT
Probe-3	GAGGGTGCTGAAATCCACCAATGTCG
	CGACATTGGTGGATTTCAGCACCCTC

Table S6 Antibodies used in this article.

Antibodies	Source	Identifier
Anti-V5	Thermo Fisher Science	Cat# R96025
Anti-GST	Proteintech	Cat# 10000-0-AP-100
Anti-His	TIANGEN	Lot# U9023
Anti-OTU7B	Beijing Protein Innovation	N/A
Anti-TRAF4	Beijing Protein Innovation	N/A
Anti-Rel1	Beijing Protein Innovation	N/A
Anti-Total Ub	Cell Signalling	Cat# 3936S
Anti-K48 Ub	Cell Signalling	Cat# 4289S
Anti-K63 Ub	Cell Signalling	Cat# 5621S
Anti-GAPDH	EASYBIO	Cat# BE0024-10
Anti-Histone H3	EASYBIO	Cat# BE3021-100
Anti-beta-Actin-HRP	EASYBIO	Cat# BE0033-100
Anti-HA	EASYBIO	Cat# BE2007-100
Anti-mouse Alexa Fluor 546	Invitrogen	Cat# A11030
Anti-rabbit Alexa Fluor 488	Invitrogen	Cat# A27034
Alexa Fluor 594	Invitrogen	Cat# A11037
Goat Anti-Mouse IgG (H&L)-HRP	EASYBIO	Cat# BE0105-100
Goat Anti-Rabbit IgG (H&L)-HRP	EASYBIO	Cat# BE0107-100
